# Effect of Probiotics on Gastrointestinal Health Through the Aryl Hydrocarbon Receptor Pathway: A Systematic Review

**DOI:** 10.3390/foods13213479

**Published:** 2024-10-30

**Authors:** Adrián De la Rosa González, Sol Guerra-Ojeda, María Alejandra Camacho-Villa, Alicia Valls, Eva Alegre, Ronald Quintero-Bernal, Patricia Martorell, Empar Chenoll, Marta Serna-García, Maria D. Mauricio, Eva Serna

**Affiliations:** 1 HARPEER Research Group, Yumbo 760001, Colombia; adrian1031@gmail.com (A.D.l.R.G.); alejacvilla@gmail.com (M.A.C.-V.); ronaldquinte29@gmail.com (R.Q.-B.); 2 Department of Physiology, Universitat de Valencia, 46010 Valencia, Spain; solanye.guerra@uv.es (S.G.-O.); alicia.valls@uv.es (A.V.); evalegrer@gmail.com (E.A.); eva.serna@uv.es (E.S.); 3 INCLIVA Biomedical Research Institute, 46010 Valencia, Spain; 4 Pain Study Group (GED), Physical Therapy School, Universidad Industrial de Santander, Bucaramanga Santander 680002, Colombia; 5 MODULAhR Group, Universitat de Valencia, 46010 Valencia, Spain; 6 Archer Daniels Midland (ADM), Nutrition, Health & Wellness, Biopolis S. L. Parc Cientific, University of Valencia, 46980 Paterna, Spain; patricia.martorell@adm.com (P.M.); maria.chenoll@adm.com (E.C.); 7 Department of Dentistry, Faculty of Health Sciences, Universidad Europea de Valencia, 46010 Valencia, Spain; marta.serna@universidadeuropea.es

**Keywords:** probiotics, aryl hydrocarbon receptor, AhR ligands, dysbiosis

## Abstract

Probiotics are living microorganisms recognized for conferring health benefits on the host by modulating the gut microbiota. They interact with various signaling pathways, including the aryl hydrocarbon receptor (AhR), which plays a crucial role in maintaining intestinal homeostasis and immune function. The activation of AhR by probiotics has been associated with benefits such as improved intestinal barrier function, reduced inflammation, and modulation of immune responses. This systematic review aims to summarize current knowledge on the signaling of AhR, mediated by probiotics in physiological conditions and gastrointestinal pathologies. We conducted a comprehensive search across databases, including PubMed and Embase, up until July 2024. Out of 163 studies screened, 18 met the inclusion criteria. Our findings revealed in healthy populations that probiotic consumption increases the production of AhR ligands promoting intestinal immune tolerance. Furthermore, in populations with gastrointestinal pathologies, probiotics ameliorated symptoms through AhR activation by Trp metabolites, leading to the upregulation of the anti-inflammatory response.

## 1. Introduction

Probiotics, according to the International Scientific Association of Prebiotics and Probiotics (ISAPP), are living microorganisms that provide health benefits to the host when consumed in adequate amounts [1]. They are commonly found in foods like yogurt, kefir, kombucha, and tempeh, or available in a dietary supplement. These products typically contain beneficial bacteria such as *Lactobacillus* spp., *Bifidobacterium* spp., *Streptococcus* spp., and others, as well as yeasts like *Saccharomyces* spp. Thus, probiotics are recognized for their protective effects on health, for example, against obesity and related cardiometabolic issues, when the administration is made in an efficacious number of viable bacteria [2]. They possess the ability to temporarily colonize the intestinal mucosa, thereby contributing to the host’s flora metabolism. However, despite our understanding of probiotics, there remains limited knowledge about their interaction with substrates or receptors. One such receptor of interest is the aryl hydrocarbon receptor (AhR), a cytoplasmic receptor and transcription factor that plays a crucial role in various physiological functions, including immune tolerance, barrier function in the gut, and maintenance of intestinal homeostasis.

**Figure 1:**
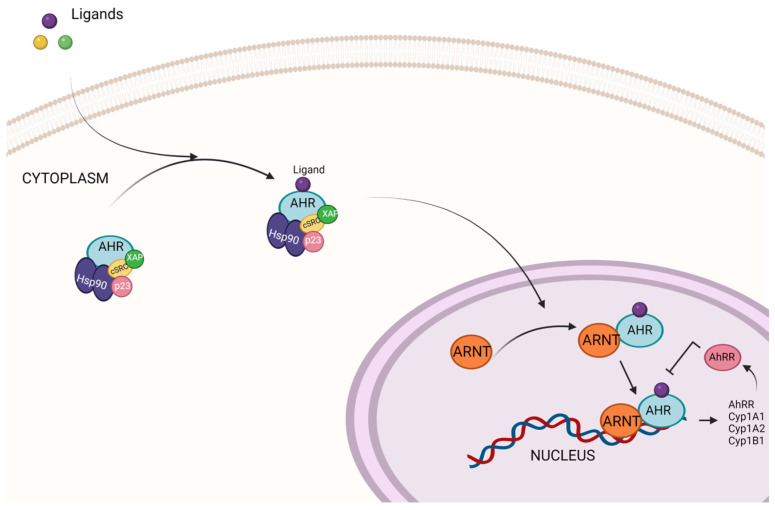
AhR signaling pathway.

The AhR is a transcription factor bHLH that is activated by both exogenous such as pollutants and endogenous ligands such as bilirubin, biliverdin, prostaglandin, indole, indole-3-acetic acid, and tryptamine [3]. Additionally, dietary ligands such as 3,3′-diindolylmethane (DIM), indole-3-carbinol (I3C), curcumin, diosmin, and urolithin A can activate the canonical AhR pathway. AhR forms a complex with HSP90 (Heat Shock Protein 90), a co-chaperone p23, an XAP molecule, and a Src tyrosine kinase in the cytosol. All these proteins facilitate the correct folding of the receptor and proper ligand recognition [4]. Therefore, AhR is stabilized in the cytoplasm through this chaperone complex until ligand binding triggers its release, transporting it to the nucleus where it heterodimerizes with the aryl hydrocarbon receptor nuclear translocator (ARNT). This heterodimer binds to DNA at AhR response elements (AHRE, 5′-GCGTG-3′), also known as dioxin response elements (DRE) or xenobiotic response elements (XRE), regulating the expression of target genes such as CYP450 [5] (Figure 1). Its best-known function is the xenobiotic response to contaminants such as TCDD but it also has different physiological and vital functions, including the maintenance and differentiation of hematopoietic stem cells, the regulation of sex hormones and reproduction, and the preservation of retinal homeostasis, and plays a crucial role in maintaining immune tolerance and barrier function in the gut [5].

In the context of the intestinal immune response, AhR plays a critical role in the maintenance of innate lymphoid cells (ILC) and intraepithelial lymphocytes (IEL), which contribute to intestinal homeostasis and barrier function. Specifically, AhR promotes the production of cytokines like IL-22 in ILC3 cells, which are essential for intestinal lymphoid follicle formation and the regulation of homeostasis [6,7,8].

Overall, modulation of the AhR signaling pathway through direct ligands or by influencing the metabolism of the gut microbiota holds potential for the treatment of various intestinal and metabolic diseases. Understanding the interaction between probiotics and AhR signaling could provide insights into novel therapeutic approaches for such conditions. For that reason, we aimed to synthesize all relevant research regarding the signaling of AhR mediated by probiotics in physiological conditions and gastrointestinal pathologies.

## 2. Methods of Searching

### 2.1. Eligibility Criteria

The following inclusion criteria were predefined according to the acronym PICOS (population, intervention, comparison, outcome, and study design) for screening papers by title and abstract: (P) patients and animal models including mice, rats and pigs; (I) administration of probiotics; (C) control group without probiotic treatment; (O) studies where the outcome was clearly and convincingly presented; and (S) studies that examined the effects of the probiotics compared to the control group. Additional inclusion criteria included (1) original, peer-reviewed studies published in English and (2) experimental studies regarding the signaling of AhR mediated by probiotics in vitro, healthy subjects, and those with gastrointestinal pathologies.

Expert opinions, editorial letters, systematic reviews, meta-analyses, case reports, and qualitative studies were excluded.

### 2.2. Search Strategy

Electronic databases of the peer-reviewed literature, including PubMed and Embase databases, were systematically searched using the PRISMA approach [9]. The key terms used in the search strategy included “Aryl hydrocarbon receptor” AND “probiotics”. The search involved all the published studies until 5 July 2024.

### 2.3. Selection Process

We used Covidence (Melbourne, VIC, Australia) to remove the duplicate studies and review all the manuscripts. Three independent reviewers (A.D.l.R., S.G.-O., and M.A.C.-V.) screened the titles and abstracts in the first round and excluded those that were irrelevant. Next, the full manuscripts of the selected papers were obtained, and those that appeared to meet the inclusion criteria were reviewed. In addition, the reference lists of the identified studies were reviewed to locate additional relevant studies. Manual searches of references were carried out by the same reviewers to identify additional articles. Any disagreement in the screening phase or data extraction phase was resolved by discussion, or, if it was necessary, by other investigators authorized to resolve conflicts (E.S. and M.D.M.).

### 2.4. Data Collection Process

All identified studies were reviewed by the same three reviewers, and information was extracted using a predetermined form (study, probiotic, sample/model, and main results).

### 2.5. Risk of Bias in Individual Studies

Three reviewers (A.D.l.R., S.G.-O., and M.A.C.-V.) independently assessed the risk of bias (RoB) of the included studies. Because different types of studies were included, we used SYRCLE’s RoB tool to assess the risk of bias in in vivo research studies [10] and an adaptation of it (see Table S1 in the Supplementary Materials) to address in vitro research. The bias information for each study was organized in a table with corresponding judgments as follows: “Yes” indicates a low risk of bias, “No” indicates a high risk of bias, and “Unclear” indicates that not enough information was reported.

## 3. Results

**Figure 2:**
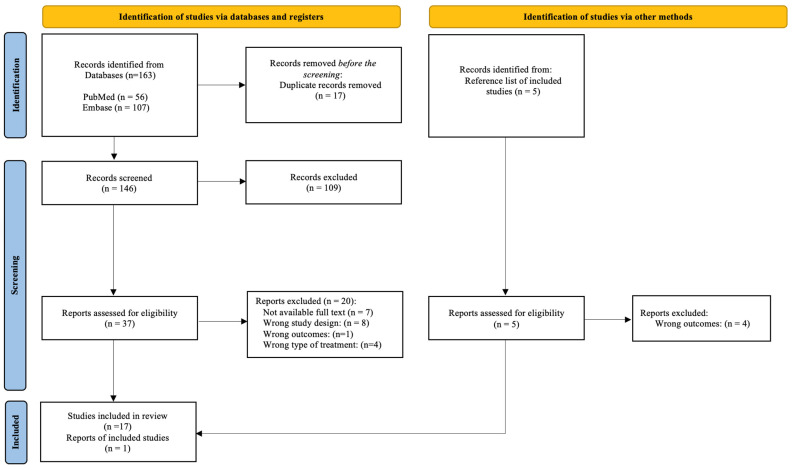
Flowchart of articles included in the systematic review.

A total of 163 articles were found from a combination of the searches. After eliminating duplications, there were 146 articles, but only 17 fulfilled the inclusion criteria. Finally, with the manual identification of studies, a total of 18 articles were included in the systematic review. The complete flowchart describing the study selection process is shown in Figure 2. References from the physiopathology and epidemiology of diseases that are included in the text are not considered in this scheme, as this is restricted to our search strategy.

### 3.1. Study Characteristics

The comprehensive study characteristics are shown in Section 3.3. All eligible studies were conducted between 2011 and 2023. Five studies (*k* = 5) investigated the effects of probiotics on AhR signaling in physiological conditions [11,12,13,14,15] and the most used strain was *Lactobacillus reuteri* (*k* = 2) [13,14]. Additionally, the in vivo models used included colon, plasma, and fecal samples from male and female C57BL/6J mice [11,13] and piglets [15].

Thirteen studies (*k* = 13) evaluated the effects of probiotics on AhR signaling in pathological conditions, including colitis (*k* = 10) [16,17,18,19,20,21,22,23,24,25], celiac disease (*k* = 1) [26], NEC (*k* = 1) [27], and intestinal barrier damage (*k* = 1) [28], with *Bifidobacterium* emerging as the most commonly used genus (*k* = 4) [22,23,24,27]. Out of the thirteen studies, two (*k* = 2/13) exclusively used in vitro models [27,29], six employed only in vivo models (*k* = 6/13) [18,20,21,22,25,26], and five (*k* = 5/13) of them included both models [16,17,19,23,24], while eight studies (*k* = 8/13) induced mice colitis with DSS [16,17,18,19,20,22,23,24].

### 3.2. Risk of Bias Assessment

Details of the risk of bias assessments for each study included in this review are presented in Table 1 and Table 2. In most studies, a high risk of selection, detection, and attrition bias was found due to problems such as sample size calculation, blinding, and incomplete outcome data.

### 3.3. Results of Individual Studies

#### 3.3.1. Relationship Between Probiotics and AhR in Physiological Conditions

Probiotics have been shown to impact the gastrointestinal system, both in vivo and in vitro. They exert their effects across various compartments of the intestine, including the intestinal microbiome, the microbe-free death zone, epithelial cells, the lamina propria rich in lymphocytes and plasma cells, the neural elements of the lamina propria, the underlying smooth muscles controlling motility, and the mesenteric lymph nodes communicating with the systemic immune system [30].

It has been shown that AhR located in this system can be activated by probiotics through their target genes. For instance, Takamura et al. [11] found that *Lactobacillus bulgaricus* OLL1181 increased mRNA expression of cytochrome P450 family 1A1 (CYP1A1) in human Caco2 cells, which was inhibited by α-naphthoflavone (αNF)*,* an AhR antagonist. Similarly, mice treated orally with a single dose of Lactobacillus bulgaricus OLL1181 showed increased CYP1A1 mRNA expression in the large intestine, with peak expression at 1–2 h and post-treatment, and gradually decreased thereafter (Table 3).

Also, it has been established that probiotics activate AhR via tryptophan (Trp) metabolites acting as ligands. In this case, a study involving 14 young men without any underlying pathologies demonstrated that the intake of probiotic yogurt fermented by *Lactobacillus delbrueckii* spp. *bulgaricus*, *Streptococcus thermophilus*, and *Lactobacillus rhamnosus* GG activated the AhR gene expression, compared to consuming unfermented acidified milk. This activation was related to certain indole compounds in dairy, including indole acid, indole-3-lactic acid (ILA), indole-3-acetic acid (IAA), and indole-3-aldehyde (IAld) [12]. Specifically, IAAld was associated with changes in AhR expression following dairy intake. However, this relationship was not straightforward and, according to the authors, could be due to external factors other than diet, which could influence fasting gene expression levels. Interestingly, AhR activation was also associated with the downregulation of inflammatory genes and was positively correlated with insulin levels after yogurt intake [12].

Liu et al. [13] found that the administration of *Lactobacillus reuteri* DSM 17938 to healthy breastfed mice changed gut microbiota and upregulated tryptophan metabolites including indoles and adenosine, which are known to activate AhR [13]. Therefore, it seems clear that AhR activation depends on Trp metabolites. However, an important question arises: can probiotics activate this receptor independently of Trp metabolism? In this regard, Özçam, M. et al. [14,31] could distinguish between two types of polyketide synthases (PKS) clusters: functionally unknown (fun) and pks, which were involved in AhR activation. PKS are secondary metabolites produced by biosynthetic gene clusters that assemble simple molecules into complex metabolites. The researchers found that in murine hepatoma cells treated with cell-free bacterial supernatants of L. reuteri R2lc and 2010 strains, the inactivation of pks prevented AhR activation in both strains.

In addition to Trp metabolism, another metabolic pathway has also been implicated in probiotic-mediated AhR activation: short-chain fatty acid (SCFA) production and signaling. Here, Xie et al. [15] showed that weaned piglets supplemented with *Lactobacillus acidophilus* and *Bacillus subtilis* significantly increased the abundance of the phylum *Firmicutes* and the *genera Clostridium*. This change in microbial diversity correlated positively with both SCFAs and tryptophan metabolism. In fact, the researchers found increased levels of butyric acid and total SCFAs, as well as protein upregulation of SCFAs receptors (GPR43 and GPR41), AhR, and IL-22. These results indicate that *Lactobacillus acidophilus* and *Bacillus subtilis* might activate AhR through the SCFAs-GPR41-AhR/HIF1α-IL-22 pathway.

In summary, the evidence suggests that probiotic strains, particularly those belonging to the *Lactobacillus* genus, can activate the AhR mainly through metabolites of Trp or SCFAs. However, further studies are needed to investigate the mechanisms of action involved in the AhR activation pathway to better understand the benefits of probiotics on gastrointestinal health.

#### 3.3.2. Relationship Between Probiotics and AhR in Gastrointestinal Pathologies

There is increasing evidence demonstrating the impact of probiotics on the AhR signaling pathway in individuals with gastrointestinal pathologies. For this reason, it is crucial to further explore these effects to elucidate the underlying mechanisms of the AhR actions in these conditions (Table 4).

##### Inflammatory Bowel Disease

Inflammatory Bowel Disease (IBD) is a chronic non-specific intestinal inflammatory disease that generally causes abdominal pain, blood stool, diarrhea, and emaciation [32]. IBD encompasses chronic inflammatory conditions such as ulcerative colitis (UC) and Crohn’s disease (CD) [33,34]. Both are linked with several pathogenic factors including environmental changes, susceptibility gene variants, bacterial dysbiosis, and a broadly dysregulated immune response [35]. In addition, diminished levels of AhR have been observed in IBD animal models and patients, suggesting its potential involvement in disease pathology [29,36,37,38]. In fact, AhR-knockout studies conducted in mice with dextran sodium sulfate (DSS)-induced colitis, showed higher levels of pro-inflammatory cytokines and increased severity of colitis compared with wild-type mice [29]. Therefore, the use of therapeutic strategies that activate the AhR could represent an alternative to mitigate the deleterious effects of IBD.

According to our review, the administration of specific probiotic strains has been shown to alleviate colitis symptoms in animal models (Table 2) through AhR activation. For instance, Cui et al. [22] found that two strains, *Bifidobacterium bifidum* (*B. bifidum*) FL-276.1 and *B. bifidum* FL-228.1, modulated the inflammatory response by downregulating the gene expression of TNF-α, IL-1β, and IL-6. These strains also mitigated colon length shortening and improved the intestinal barrier by upregulating gene expressions of tight junctions such as claudin-4, occluding, and zonula occludens-1 (ZO-1) [22]. Since supplementation with both strains increased the gene expression of AhR and C1P1A1, it was estimated that DSS-induced colitis may have improved through the AhR pathway. Furthermore, a supplementary report conducted by the same group, using the same strains, examined the effects of both the whole course (from the beginning of the experiment) and partial intervention (after the DSS induction started). The study demonstrated that only whole course intervention with both strains increased the serum content of ILA and improved colon length. Furthermore, total and partial interventions enhanced the epithelial barrier function and reduced intestinal inflammation through the AhR/Nfr2/NLRP3 pathway and their target genes CYP1A1 and NQO1 [23].

Park et al. [24] investigated the mechanism of action of *Bifidobacterium breve CBT BR3* (*B. breve*) in vitro and in vivo. In this study, colitis was induced in mice using DSS and dinitrobenzene sulfonic acid (DNBS), and Caco2 and HT29-Lucia^TM^ cell lines were also utilized. Researchers found that in both models, *B. breve* preserved colon length, restored globet cell function, and elevated mRNA expression of mucin5, IL-10, and IL-22. Moreover, *B. breve* protected inflammation-induced epithelial cell permeability in Caco2 cells by modulating the mRNA expression of IL1β, mucin2, occludin, and REG3G, probably through AhR induction.

*Lactobacillus reuteri* is another strain that significantly modulates AhR to alleviate colitis symptoms. Supplementation of *Lactobacillus reuteri* has been shown to stimulate interleukin (IL)-22, a cytokine regulated by AhR, and rescue susceptibility to DSS-induced colitis by preserving body weight and reducing colon length, intestinal structure damage, and pro-inflammatory cytokines such as TNF-α and IL1β [19]. Furthermore, metabolites derived from tryptophan in *Lactobacillus reuteri*, such as IAA and IAld, have been found to restore IL-22 production [16]. The study by Zalante et al. [16] also confirmed the role of the AhR pathway in the effects of *Lactobacillus reuteri*, as they observed that the reduction in colitis symptoms did not occur in AhR^−/−^ mice. Moreover, Hou et al. [19] showed that increased production of IL-22 in lamina propria lymphocytes (LPLs) challenged with IAA was inhibited by the AhR antagonist CH-223191. In fact, they suggest that *Lactobacillus reuteri* induced LPLs to secrete IL-22 through AhR and then activated STAT3 phosphorylation to counteract epithelial damage in DSS-induced colitis mice.

The effects of *Lactobacillus* strains have also been studied in Card9^−/−^ (a susceptibility gene for IBD) mice exposed to DSS. After treatment with three Lactobacillus strains (*Lactobacillus murinus*, *Lactobacillus reuteri*, and *Lactobacillus taiwanensis*), IL-22 production was rescued, and intestinal inflammation was attenuated through AhR activation [18]. This result may be related to the impaired production of IAA in Card9^−/−^ mice, known to promote local IL-22. *Lactobacillus acidophilus* or its metabolite, indole-3-lactic acid (ILA), was also probed to relieve inflammatory status through AhR in DSS-induced colitis cesarean section offspring [25].

Emerging evidence has also revealed that *Akkermansia muciniphila* (*Akk*), a Gram-negative anaerobic bacterium improves the clinical parameters of colitis, including spleen weight, colon inflammation index, and colon histological score. Gu et al. [20] found that the disease disrupts metabolic serum profiling in humans and mice by altering metabolites involved in the AhR pathway. However, they found that pasteurized Akk supplementation increased serum levels of endogenous ligands of AhR derived from Trp metabolism (i.e., IAld, 5HIAA) in mice with DSS-induced colitis. Considering that colitis altered 300 genes, RNA sequencing showed that supplementation with Akk and a specific protein isolated from its outer membrane (Amuc_1100) upregulated 123 and 28 genes involved in Trp metabolism, respectively. Finally, live and pasteurized Akk and Amuc_100 alleviate colonic inflammation, restoring the downregulated AhR target genes such as CYP1A1, IL-10 and IL-22 [20]. Additionally, Li et al. [21] found that *B. thetaiotaomicrono bacteroides*, a strain belonging to the Bacteroides genus, alleviated DSS-associated intestinal inflammation by increasing the serum concentration of IL-10 in mice. Moreover, strain administration elevated Trp metabolites and the AhR expression, reducing colon damage.

In addition to the Trp metabolites produced by probiotics acting as AhR activators to mitigate colitis, the involvement of other bacterial metabolites has also been observed. For instance, 1,4-dihydroxy-2-naphthoic acid (DHNA), a precursor of vitamin K2 and abundantly produced by *Propionibacterium freudenreichii* ET-3, was able to increase CYP1A1 gene expression in Caco2 cells, and in the mouse intestine, which was inhibited by CH-223191, an AhR antagonist [17]. This AhR-dependent effect was confirmed in AhR^−/−^ mice. Also, DHNA reduced DSS-induced colitis markers, such as inflammatory histological scores and TNFα expression, which again was inhibited by CH-223191 [17].

Impaired intestinal barrier and increased intestinal permeability are closely related to IBD. In this regard, a previous study from Wang’s lab [28] explored whether the *Lactiplantibacillus plantarum* (*L. plantarum*) DPUL-S164 intervention, in human colon adenocarcinoma cells (HT29), alleviates intestinal barrier damage and inflammation. Treatment with the strain increased the expression of AhR mRNA and its target gene CYP1A1 in HT29 cells. It was also able to restore AhR, Nrf2, and CYP1A1 mRNA expression after LPS-induced damage. Finally, the study also reported that the inflammatory response was reduced by modulating the expression and mRNA content of IL-10 and TNFα.

In summary, these studies demonstrated that probiotics like, *Bifidobacterium bifidum*, *Bifidobacterium breve*, *Propioni-bacterium freudenreichii*, *Lactobacillus reuteri* D8, *Lactobacilli* spp., *Lactobacillus acidophilus*, and *Akkermansia muciniphilia* produce Trp metabolites (i.e., ILA, 5HIAA, and IAld). These ligands upregulate mRNA expression of genes involved in the promotion of epithelial regeneration and reducing intestinal inflammation, via AhR. Thus, the administration of these strains is useful to attenuate clinical parameters of colitis.

##### Celiac Disease

Celiac disease (CD) is an autoimmune inflammatory enteropathy triggered by the intake of gluten and related prolamins in genetically susceptible individuals [39]. The pathology is characterized by systemic symptoms related to malabsorption and immune activation, auto-antibodies to tissue transglutaminase (TTG), human leukocyte antigen (HLA)-DQ2 or HLA-DQ8, and small intestine damages [39,40]. These alterations disrupt the body’s ability to uptake nutrients from food. The development of intestinal inflammation in CD has been linked to reduced expression of AhR and its ligands, which are produced by the gut microbiota [41]. Furthermore, reduced key bacterial groups that are known to metabolize tryptophan into AhR ligands, along with impaired metabolic function, have been observed in CD patients.

Recently, Lamas et al. [26] identified a potential pathogenic mechanism related to the impaired production of AhR ligands by the gut microbiota in CD. They used non-obese diabetic mice expressing the DQ8 celiac disease susceptibility gene with or without gluten exposure and proved three interventions to activate the intestinal AhR pathway: (i) treatment with a low- or high-Trp diet before and after gluten exposure, (ii) oral supplementation with AhR ligand-producing *Lactobacillus* during gluten treatment, or (iii) treatment with either an AhR agonist or vehicle during gluten treatment. Results from this study indicated that, compared to a low Trp diet, the high Trp diet shifted gut microbiota composition, leading to a higher abundance of *Lactobacillus* and *Ruminococcus gnavus*, which are known AhR ligand producers. In addition, mice fed the high Trp diet showed lower Fecal lipocalin-2 content, duodenal IL6 expression, intraepithelial lymphocyte counts, ion transport, and paracellular permeability than low fed Trp diet, which are key measurements for diagnosing CD. In contrast, researchers found that the feces of mice fed with the low Trp diet showed lower levels of kynurenine, a Trp metabolite implicated in chronic inflammation [42,43]. These results suggest that a high-supplemented Trp diet can reverse gluten-induced immunopathology in NOD/DQ8 mice by preserving gut microbiota and highlighting the gut microbiota’s role in modulating the AhR pathway in CD.

##### Necrotizing Enterocolitis

Necrotizing enterocolitis (NEC), a multifactorial and not completely understood gastrointestinal disease primarily affecting premature neonates [44], is characterized by dysregulated inflammatory responses to luminal bacteria [45]. Ischemic necrosis affects both the small and large intestines, leading to the translocation of enteric organisms into the circulation, often resulting in overwhelming sepsis and death [46,47]. Despite breastfeeding being identified as a protective factor against NEC, mortality rates associated with the condition vary widely, ranging from 15% to 45% [48]. Survivors of NEC frequently experience severe sequelae such as growth restriction, short-bowel syndrome, and neurological deficits [49,50].

Its pathophysiology involves mesenteric vasculature ischemia, disrupted in-utero signaling, and the intricate interplay among dysbiotic enteric microbes in the premature gut [44,51,52]. This interplay leads to impaired immune function and epithelial cell death through apoptosis, autophagy, and necroptosis. These signaling pathways are initiated in response to toll-like receptor 4 (TLR4) on the intestinal epithelium [44,53,54,55,56,57].

Lipopolysaccharide (LPS) is the main endotoxin in the cell walls of Gram-negative bacteria. TLR4 is activated by LPS on the intestinal epithelium, triggering a shift from a developmental to an inflammatory role and inducing NEC [58].

Recently findings suggested that during pregnancy, administration of a diet rich in the AhR like the ligand indole-3-carbinole (I3C), or of breast milk, prevented NEC in newborn mice by reducing TLR4 signaling in the gut through the activation of AhR. Additionally, AhR activation was shown to reduce inflammation induced by LPS in the intestinal epithelium. Lu et al. [59] demonstrated the role of AhR in intestinal samples from premature infants undergoing NEC surgery. As happened in mice experiments, higher signaling of TLR4 induced by LPS was reduced by AhR activation through IC3 treatment in vitro, suggesting AhR’s significant role in mitigating NEC pathology.

On the other hand, the administration of probiotics in the prevention of NEC has also been studied. Several randomized controlled trials (RCTs), systematic reviews and meta-analyses of RCTs, and experimental and observational studies have consistently demonstrated the preventive action of probiotics in preterm babies against NEC [60,61,62,63]. A 2017 meta-analysis, which included 25 RCTs and 7435 neonates, found that the supplementation of multiple-strain probiotics significantly reduced NEC incidence [64]. Zhang et al. [65] found that the use of *Bifidobacterium* and *Lactobacillus* supplementation reduced the incidence of NEC compared to placebo [65].

The molecular mechanisms underlying the effect of probiotics on NEC remain unclear [66,67,68]. However, one potential mechanism involves the AhR molecular pathway. Meng et al. [27] demonstrated that ILA present in large amounts in breastmilk, and produced by *Bifidobacterium longum* subspecies *infantis*, generated an anti-inflammatory effect in immature human NEC enterocytes. ILA was found to mediate its effects through AhR, reducing the IL1β and IL-8 secretion, thereby mitigating excessive inflammation in the premature intestine and preventing NEC [27].

## 4. Discussion

This review highlights the beneficial effects of probiotic administration on host health, particularly through mechanisms involving the production of Trp metabolites and activation of the AhR signaling pathway. While current evidence suggests probiotics can positively influence health in both healthy individuals and populations with gastrointestinal disorders, additional research is required to elucidate the specific roles of AhR signaling in both human and animal models.

The main findings from this review suggest the beneficial effects of probiotic administration on host health. Although the positive effects of probiotics in healthy populations are mediated through the production of Trp metabolites, more research in both human and animal models to study the AhR signaling pathway is necessary.

Furthermore, in populations with gastrointestinal pathologies, studies have shown a favorable effect of probiotics in the clinical parameters of UC, CD, and NEC through AhR activation by Trp metabolites (i.e., ILA, 5HIAA, IAld, and DHNA). The amelioration of symptoms in these pathologies is primarily mediated through the upregulation of the anti-inflammatory response, including increased expression of IL-22 and target genes (i.e CYP1A1, NQO1, and Nfr2) and simultaneous downregulation of TNFα, IL10, and IL-8. Commonly used microorganisms encompass *Lactobacillus bulgaricus*, *Streptococcus thermophilus*, *Lactobacillus acidophilus*, *Akkermansia muciniphilia*, *Lactiplantibacillus plantarum* and various species of *Bifidobacterium*.

Moreover, the potential role of the AhR pathway could extend beyond gastrointestinal health. The same mechanisms by which probiotics influence the gut could also impact other organs and systems, including the brain, through the gut–brain axis. Thus, probiotics and their metabolites could have broader systemic effects, warranting further investigation.

Finally, a future challenge in the field of probiotics and symbiotics is based on new clinical trials that explore the relationship between probiotics and AhR. This connection carries promising implications for future therapeutic interventions in the treatment of other gastrointestinal and metabolic diseases in animal models and humans. This entails identifying the strains, mechanisms of action, and optimal doses necessary for the treatment of these pathologies.

## Figures and Tables

**Table 1:** Risk of bias for in vitro studies (Modified SYRCLE’s RoB tool).

Study	Item 1	Item 2	Item 3	Item 4	Item 5	Item 6	Item 7	Item 8	Item 9	Item 10
Takamura, et al., 2011 [11]	No	Yes	Yes	Yes	Yes	Yes	No	Yes	No	Unclear
Ozçam et al., 2019 [14]	No	Yes	Yes	Yes	Unclear	Unclear	No	No	Unclear	Yes
Zelante et al., 2013 [16]	No	Yes	Yes	Yes	Yes	Yes	No	Yes	Unclear	Unclear
Fukumoto et al., 2014 [17]	No	Yes	Yes	Unclear	Yes	Unclear	No	Unclear	Yes	No
Hou et al., 2018 [19]	No	Yes	Yes	Yes	Yes	Yes	No	Yes	Yes	Yes
Meng et al., 2020 [27]	No	Yes	Yes	Yes	Yes	Yes	No	Yes	Yes	Yes
Cui et al., 2023 [23]	No	Yes	Yes	Yes	Yes	Yes	No	Yes	Unclear	Yes
Park et al., 2023 [24]	No	Yes	Yes	No	Yes	Yes	No	Yes	Yes	Yes
Wang et al., 2023 [28]	No	Yes	Yes	Yes	Yes	Yes	No	No	Yes	Yes

**Table 2:** Risk of bias for in vivo studies (SYRCLE’s RoB tool).

Study	Item 1	Item 2	Item 3	Item 4	Item 5	Item 6	Item 7	Item 8	Item 9	Item 10
Takamura et al., 2011 [11]	No	No	No	No	No	No	No	Unclear	No	Unclear
Liu et al., 2019 [13]	No	Yes	Yes	Yes	Yes	Yes	No	Unclear	Yes	Yes
Xie Z et al., 2022 [15]	No	Yes	Yes	Yes	Yes	Yes	No	No	Unclear	Yes
Zelante et al., 2013 [16]	No	Yes	Yes	Yes	Yes	No	No	No	Yes	Yes
Fukumoto et al., 2014 [17]	No	Unclear	Unclear	Unclear	Yes	Unclear	No	No	Yes	No
Lamas et al., 2016 [18]	Yes	Yes	Yes	Yes	No	Yes	Yes	Yes	Yes	Yes
Hou et al., 2018 [19]	No	Yes	Yes	Yes	Yes	Unclear	No	Yes	Yes	Yes
Lamas et al., 2020 [26]	No	Yes	Yes	Yes	Yes	Yes	Yes	Yes	Yes	No
Gu et al., 2021 [20]	No	Yes	Yes	Yes	Yes	Unclear	No	Unclear	Yes	Yes
Li et al., 2021 [21]	No	Yes	Yes	Yes	Yes	Yes	No	No	Yes	Yes
Cui et al., 2022 [22]	No	Yes	Yes	Yes	Yes	Yes	No	No	Yes	Yes
Cui et al., 2023 [23]	No	Yes	Yes	Yes	Yes	Yes	No	No	Yes	Yes
Park et al., 2023 [24]	No	Yes	Yes	Yes	Yes	Yes	No	No	Yes	Yes
Xia et al., 2023 [25]	No	Yes	Yes	Yes	Yes	Yes	No	No	Yes	Yes

**Table 3:** Studies selected on the relationship of probiotics and their effect on AhR signaling pathway in physiological conditions.

Study	Probiotic	Sample/Model	Main Results
Takamura et al., 2011 [11]	*Lactobacillus bulgaricus* OLL1181 (1 × 10^9^ CFU/mL)	Human Caco2 cells Colon from female C57BL/6 mice Age = 4 to 6 weeks old	*L. bulgaricus* induces mRNA expression of CYP1A1 in Caco2 and mouse colon. CYP1A1 expression in the mouse colon peaked at 1–2 h and gradually decreased after treatment with *L. bulgaricus*.
Burton et al., 2018 [12]	Probiotic yogurt (*Lactobacillus. delbrueckii* spp. *Bulgaricus*, *S. thermophilus* and *L. rhamnosus GG*)	Serum, plasma, and whole blood collected postprandially from young male patients Age = 24.6 ± 4.7 years	Probiotics increase the concentration of IAld, a ligand of AhR. Changes in circulating insulin correlate positively with changes in AhR expression 2 h after yogurt intake. AhR contributes significantly to the downregulation of inflammatory and glycolytic genes (i.e., CSF1, RHOG).
Liu et al., 2019 [13]	*Lactobacillus reuteri* DSM 17938 (10^7^ CFU/day)	Plasma and fecal samples from female and male C57BL/6J mice Age = 8 days to 2 weeks	Upregulates N-acetyltriptophan and Trp metabolites. Changes bacterial diversity: increases Firmicutes, decreases Bacteroidetes.
Ozçam et al., 2019 [14]	*Lactobacillus reuteri* R2lc and 2010	Murine hepatoma cell line H1L6.1c3	*L. reuteri* R2lc and 2010 activate AhR through polyketide synthase (PKS) cluster genes.
Xie Z et al., 2022 [15]	*Lactobacillus acidophilus* and *Bacillus subtilis* (10^6^ CFU/g)	Colon and colonic contents from piglets Age = 28 days	Combination of *L. acidophilus* and *B. subtilis* significantly increases the levels of butyric acid and total SCFAs, and protein expression of SCFAs receptors, AhR, HIF1α, and IL-22.

AhR: aromatic hydrocarbon receptor; CFU: colony forming units; GVHD: acute graft-versus-host disease; IAld: indole-3-Aldehyde; Trp: tryptophan; SCFA: short-chain fatty acids.

**Table 4:** Studies selected on the relationship of probiotics and their effect on AhR signaling pathway in pathological conditions.

Study	Pathology	Probiotic	Sample/Model	Main Results
Zelante et al., 2013 [16]	Colitis	*Lactobacillus reuteri* (10^8^ CFU)	Stomachs from C57BL/6 Colon and colonic NKp46^+^ cells from C57BL/6 and AhR^−/−^ mice with DSS-induced colitis Age = 8 to 10 weeks old	IAld is detected in ex vivo cultures of stomach exposed to *L. reuteri.* IAld restores IL-22 production and ameliorates colitis in colon from C57BL/6 but not from AhR^−/−^ mice. IAld induces IL-22 production by colonic NKp46^+^ cells in C57BL/6 mice.
Fukumoto et al., 2014 [17]	Colitis	DHNA (derived of *Propionibacterium freudenreichii* ET-3 strain)	Human Caco2 cells Small intestine and large intestine from male C57BL/6, AhR^−/−^ and DSS-induced colitis mice Age = 6 to 8 weeks old	DHNA increases CYP1A1 mRNA expression in Caco2 cells and small intestine and large intestine from C57BL/6 mice but not from AhR^−/−^ mice. This effect was inhibited by CH-223191, an AhR antagonist. DHNA reduces colon shrinkage, histological inflammatory scores, and myeloperoxidase and tumor necrosis factor-α expression in colon from DSS-induced colitis mice. This effect was inhibited by CH-223191.
Lamas et al., 2016 [18]	Colitis	*Lactobacillus. murinus* CNCM I-5020 *Lactobacillus. reuteri* CNCM I-5022 *Lactobacillus taiwanensis* CNCM I-5019	Colon from male C57BL/6J mice and Card9^−/−^ with DSS-induced colitis Age = 8 weeks old	Lactobacillus strains promote recovery of DSS-induced colitis and increase IL-22 expression and AhR activity in Card9^−/−^.
Hou et al., 2018 [19]	Colitis	*Lactobacillus* *reuteri D8*	Jejunum and colon from C57BL/6 mice with DSS-induced colitis Age = 4 weeks old Co-cultured system of mouse intestinal organoids with lamina propria lymphocytes from small intestine	*L. reuteri D8* ameliorates DSS-induced colitis by reducing colon length, intestinal structure damage, TNF-α, and IL1β protein expression, and enhancing IL-22 production. *L. reuteri D8* upregulates IL-22 in mice and co-cultured models. IAA increases IL-22 in co-cultured model. The AhR inhibitor CH-223191 inhibits IL-22 production induced by *L. reuteri D8* or IAA. *L. reuteri D8* and IL-22 induce phosphorylation of STAT3, a transcriptional factor that promotes proliferation of intestinal epithelial.
Lamas et al., 2020 [26]	Celiac disease	*Lactobacillus reuteri* CNCM-I5022 and CNCM-I5429	Duodenum, feces, and plasma from male and female gluten-treated NOD/DQ8 mice Age = 8 to 12 weeks old	Activation is beneficial. Mice fed a Trp-enriched diet exhibit greater expression of CYP1A1. Fecal lipocalin-2 and duodenal IL-6 expression are lower in high tryptophan-fed mice. *Lactobacillus reuteri* significantly increases the capacity of the small intestinal microbiota to activate AhR in gluten-treated NOD/DQ8 mice fed an enriched tryptophan diet. *Lactobacillus reuteri* enhances villus-to-crypt ratio and increases plasmatic concentration of AhR ligands in gluten-treated NOD/DQ8 mice fed an enriched tryptophan diet.
Meng et al., 2020 [27]	Necrotizing enterocolitis (NEC)	*Bifidobacterium longum* subsp. *Infantis* (*B. infantis*)	H4 cells Enterocytes with NEC from the viable margins of resected ileal NEC tissues from a NEC neonate at 25-week gestation Human immature intestinal organoids from gestational age 15 and 22 weeks are therapeutically aborted	ILA reduces inflammation in enterocytes, H4 cells, samples of small intestine from patients with NEC, and other immature enterocytes. ILA, a breakdown molecule of breastmilk Trp metabolized by *B. infantis* reduces the IL-8 responses by AhR activation in H4 cells.
Gu et al., 2021 [20]	Colitis	*Akkermansia muciniphila* (*Akk*)	Plasma and colon of male C57BL/6J mice with DSS-induced colitis Age = 6–8 weeks old	Pasteurized Akk increases serum levels of AhR pathway ligands (i.e., IAA and IA) in mice with DSS-induced colitis. Live Akk, pasteurized Akk and Amuc_1100 restore the downregulation of AhR target genes (i.e., CYP1A1, IL-10, and IL-22) in the colon tissue of mice with DSS-induced colitis.
Li et al., 2021 [21]	Colitis	*Bacteroides thetaiotaomicron* (*B. thetaiotaomicron*)	Colon from male KM mice Age = 6–8 weeks old Weight= 20 ± 2 g	*B. thetaiotaomicron* protects against DSS-associated intestinal inflammation by increasing serum concentration of IL-10 along with reducing IFN-γ and IL-17 in mice. *B. thetaiotaomicron* administration increases Trp metabolites levels IAA and IPA along with the AhR expression in colon. *B. thetaiotaomicron* reduces the expression of CYP1A1 compared to DSS model group. *B. thetaiotaomicron* diminishes colon damage as assessed by histopathological scores despite elevated colon length.
Cui et al., 2022 [22]	Colitis	*Bifidobacterium bifidum* FL-276.1 and FL-228.1	Colon from C57BL/6N male mice with DSS-induced colitis Age = 4 weeks old	Both strains ameliorate DSS-induced colitis avoiding body weight loss and intestinal barrier damage in mice. Both strains upregulate gene expression of zonula occludens-1, claudin-4, occludin, and mucin 2, and downregulated expression of TNF-α, IL-1β, and IL-6. Also, they upregulate AhR protein expression and target gene C1P1A1.
Cui et al., 2023 [23]	Colitis	*Bifidobacterium bifidum FL-276.1* and *FL-228.1*	Caco2 cells exposed to lipopolysaccharide (LPS) Serum and colon of male C57BL/6N mice with DSS-induced colitis Age = 4 weeks old	Total and partial administration of *Bifidobacterium bifidum* FL-276.1 and *Bifidobacterium bifidum* FL-228.1 ameliorate DSS-induced colitis by preventing weight loss and reducing the disease activity index. Oral administration of two strains relieves DSS-induced colitis by reducing mRNA expression and content of TNF-α, IL-1β, and IL-16 in colonic tissue in both total and partial interventions. Whole course or partial intervention of both strains increases the gene and protein content of AhR and NRF2. Also, they increase AhR and NRF2 target genes such as CYP1A1 and NQO1. Whole course intervention of both strains increases serum content of ILA, an AhR ligand. ILA treatment upregulates the gene and protein expression of zonula occludens-1, claudin-4, occludin, and NQO1 in Caco2 cells exposed to LPS. This upregulation is reversed with the AhR antagonist CH-223191.
Park et al., 2023 [24]	Colitis	*Bifidobacterium breve CBT BR3* (*B. breve*)	Colon of C57BL/6 male mice with DSS or dinitrobenzene sulfonic acid (DNBS)-induced colitis Age = 8 weeks old Caco2 and HT29-Lucia^TM^ cells exposed to TNF-α and H_2_O_2_	*B. breve* ameliorates DSS-induced colitis in mice by reducing disease activity index, preserving body weight and colon length. *B. breve* ameliorates DNBS-induced colitis in mice by preserving colon length and increasing the number of globet cells and mucus thickness. Heat-killed cells of *B. breve* improve permeability in Caco2 cells challenged with TNF-α. Heat-killed cells of *B. breve* downregulate gene expression of *IL1B*, and increase the gene expressions of MUC2, occluding, and REG3g in H_2_O_2_-treated Caco2 cells. Heat-killed cells of *B. breve* significantly elevate AhR promoter activity in HT29-Lucia^TM^ AhR cells.
Wang et al., 2023 [28]	Intestinal barrier damage	*Lactiplantibacillus plantarum* (*L. plantarum*) *DPUL-S164* Isolated from human feces.	HT-29 cells with LPS-induced barrier damage	*L. plantarum DPUL-S164-TM* reduces inflammatory status by increasing the mRNA expression and content of IL -10 and reducing the mRNA expression and content of TNFα. *L. plantarum DPUL-S164-TM* mitigates LPS-induced barrier damage in HT-29 cells by enhancing the mRNA and protein expression of tight junction proteins occludin and claudin1. *L. plantarum DPUL-S164* rescues AhR, Nrf2, CYP1A1, and HO-1 mRNA expression and AhR, CYP1A1, and HO-1 protein expression after addition of LPS in HT-29 cells. *L. plantarum DPUL-S164* activates the AhR and Nrf2 signaling pathways and suppresses the NF-κB signaling pathway in HT-29 cells. *L. plantarum* DPUL-S164-TM increases ILA content.
Xia et al., 2023 [25]	Colitis	*Lactobacillus acidophilus*	Colon of mice with DSS-induced colitis divided into two groups: Cesarean section (CS); N = 6 Vaginal delivery (VD); N = 6 Age = 6–8 weeks old	*Lactobacillus acidophilus* relieves DSS-induced mice colitis by preserving body weight, reducing disease activity index and colon length in CS mice. CS colitis mice with probiotic treatment alleviate inflammation by reducing IL1β and TNFα levels. Moreover, the strain alleviates the decreased expression of IL-22 in the colon of both CS and VD mice. ILA increases IL-22, CYP1A1, Fmo2, and Kit mRNA expressions on ILC3 of CS mice through AhR.

Abbreviations: AhR^−/−^: homozygous for deletion of the AhR gene; AhR^+/−^: heterozygous for deletion of the AhR gene; Caco2: colonic adenocarcinoma cells; CARD9: caspase recruitment domain-containing protein 9; CFU: colony forming units; COX2: cyclooxygenase; CYP1A1: cytochrome P450 family 1A1; DHNA: 1,4-dihydroxy-2-naphthoic acid; DNBS: dinitrobenzene sulfonic acid; DSS: dextran sodium sulfate; GPX2: glutathione peroxidase 2; IA: indole acrylic acid; IAA: indole-3-acetic acid; IAld: indole-3-Aldehyde; IELs: intraepithelial lymphocytes; ILA: indole-3-lactic acid; IL1β: inteleukin-1 beta; IL-6: interleukin 6; IL-8: interleukin 8; IL-10: interleukin 10; IL-22: interleukin 22; IPA: indole propionic acid; NOD: non-obese diabetic; NQO1: NAD(P)H quinone dehydrogenase 1; NF-κB: nuclear factor kappa-light chain enhancer of activated B cells; NR: not reported; Nrf2: nuclear factor erythroid 2-related factor 2; PGE2: prostaglandin E_2_; REG3G: regenerating islet-derived protein 3-gamma; STAT3: signal transducer and activator of transcription 3; TNF-α: tumor necrosis factor-alpha; Trp: tryptophan.

## Data Availability

The original contributions presented in this study are included in the article/Supplementary Materials. Further inquiries can be directed to the corresponding author.

## Supplementary Materials

The following supporting information can be downloaded at: https://www.mdpi.com/article/10.3390/foods13213479/s1, Table S1: Modified SYRCLE’s RoB tool.
