# Supplementary material for: Effect of Probiotics on Gastrointestinal Health Through the Aryl Hydrocarbon Receptor Pathway: A Systematic Review
Source: Foods. 2024 Oct 30;13(21):3479. doi: 10.3390/foods13213479 (PMC11545787; doi:10.3390/foods13213479)
Supplement: Supplementary file 1 [file foods-13-03479-s001.zip › foods-3266682-supplementary.pdf]

Supplementary Methods

Modified SYRCLE's RoB tool. The *SYRCLE's RoB tool,* designed to assess bias in animal intervention studies, was adjusted for application to in vitro research. Selection bias comes from possible differences between the chosen cell lines that may impact the study outcomes. The performance bias was reformulated to highlight the variability of the intervention. For detection bias, we focused on transparency and objectivity of outcome assessment. Finally, the assessment of other biases involved the presentation of data, transparency in the declaration of conflicts of interest and funding sources, and the availability of data for independent verification The resulting RoB tool for in vitro studies included in our review contains 10 entries, as shown in Table S1.

Table S1. *Modified SYRCLE's RoB tool*

Item	Type of bias	Domain	Description of domain	Review authors judgment
1	Selection bias	Sample size calculation	Details of the sample size calculation method (software, formula and parameters used to calculate the sample size) should be clearly indicated.	Was the sample size properly calculated and applied?
2	Selection bias	Baseline characteristics	Details of cell lines used	Is it clear whether the cell lines used are primary cell cultures or continuous cell lines from the gastrointestinal system?
3	Performance bias	Detailed explanation of intervention	The intervention procedure must be clearly indicated, indicating the concentration, the vehicle and the incubation time of the substances administered	Were interventions administered to cells in a consistent and standardized manner throughout the experiment?
4	Performance bias	Detailed explanation of culture conditions	Provide any information relating to culture conditions	Are culture conditions comparable between different studies or groups within studies?
5	Detection bias	Details of comparison group	Details of comparison group (positive control, negative control, or standard) should be clearly specified.	were appropriate controls included?
6	Detection bias	Method of measurement of outcome	The procedure and rationale for the choice of method, and how the results were evaluated should be clearly stated	Were the statistical analysis clearly specified?
7	Detection bias	Blinding	Details on all measures used, if any, to prevent outcome assessors from knowing which intervention each cell culture received. Provide any information regarding the effectiveness of the planned blinding	Was the outcome assessor blinded? (*)
8	Attrition bias	Incomplete outcome data	Describe the completeness of outcome data for each main outcome, including attrition and exclusions from the analysis	Were incomplete outcome data adequately addressed? (*)
9	Reporting bias	Selective outcome reporting	State how selective outcome reporting was examined and what was found	Are reports of the study free of selective outcome reporting? (*)
10	Other	Other sources of bias	State any important concerns about bias not covered by other domains in the tool	Was the study apparently free of other problems that could result in high risk of bias? (*)

* Items consistent with the items in the SYRCLE tool.
